# Interim Analysis of Impact of Adding Low Dose Pulmonary Radiotherapy to Moderate COVID-19 Pneumonia Patients: IMpaCt-RT Study

**DOI:** 10.3389/fonc.2022.822902

**Published:** 2022-03-29

**Authors:** Pritanjali Singh, Avik Mandal, Dharmendra Singh, Subhash Kumar, Amarjeet Kumar, Amrita Rakesh, Rakesh Ranjan, Manika Verma, Deependra Kumar Rai, Divendu Bhushan, Abhishek Shankar, Arkaprava Sinha, Rohit Saini, Arijit Saha, Ashwin Thovarayi, Anindya Kumar Baral, Samyak Chauhan, Rajhans Kumar, Priya Kakoty, Bithika Modak, Alok Ranjan

**Affiliations:** ^1^ Department of Radiation Oncology, All India Institute of Medical Sciences, Patna, India; ^2^ Department of Radio-diagnosis, All India Institute of Medical Sciences, Patna, India; ^3^ Department of Anaesthesiology, All India Institute of Medical Sciences, Patna, India; ^4^ Department of Pulmonary Medicine, All India Institute of Medical Sciences, Patna, India; ^5^ Department of General Medicine, All India Institute of Medical Sciences, Patna, India; ^6^ Department of Community and Family Medicine, All India Institute of Medical Sciences, Patna, India

**Keywords:** COVID-19, low dose radiation, CT severity score, NEWS 2, COVID pneumonia

## Abstract

**Background:**

Treatment for coronavirus disease 2019 (COVID-19) pneumonia remains largely supportive till date and multiple clinical trials took place within the short span of time to evaluate the role of investigational therapies. The anti-inflammatory effect of low dose whole lung radiation in treating pneumonia has been documented earlier. This clinical trial analyzed the effect of low dose radiation therapy (LDRT) in a moderately affected COVID-19 pneumonia patient cohort and has evaluated its effect in stopping the conversion of moderate disease into severe disease.

**Methods:**

Patients with moderate COVID-19 pneumonia as characterized by the Ministry of Health and Family Welfare (MOHFW), Government of India, were randomized (1:1) to low dose whole lung radiation versus no radiation. All treatment of patients was concurrently being given as per institutional protocol. Patients were followed up with clinical and laboratory parameters monitored on Days 1, 3, 7, and 14. Computed tomography scan (CT scan) of thorax was performed on Days 1 and 7. Patients were evaluated for conversion of moderate into severe disease as per National Early Warning Score-2 (NEWS-2 score) as the primary end point. The secondary endpoints included changes in ratio between peripheral capillary oxygen saturation and fraction of inspired oxygen (SpO2/FiO2), biochemical markers, 25-point CT severity score, and radiation induced acute pulmonary toxicities.

**Findings:**

At the interim analysis, there were seven patients in the radiation arm and six in the control. A whole lung LDRT improved the outcome of SpO2/FiO2 at Day 3; however it did not convert into a statistically significant improvement for the NEWS-2 score. The serum levels of LDH, CRP, Ferritin and D-dimer were significantly reduced on 14 days in the LDRT arm in comparison to the baseline value but were not significant between the two groups.

**Interpretation:**

LDRT seems to have the potential to prevent moderate COVID-19 pneumonia from a deteriorating to severe category. However, further randomized clinical trial with an adequate number of such patients is warranted to establish the definitive role of LDRT in the management of COVID-19 pneumonia.

**Funding:**

An intramural research project bearing code: I-27/621, was sanctioned from the All India Institute of Medical Sciences, Patna, India.

**Clinical Trial Registration:**

Clinical Trials Registry-India (CTRI/2021/06/033912, 25th May 2021) ctri.nic.in/Clinicaltrials/login.php

**Graphical Abstract:**
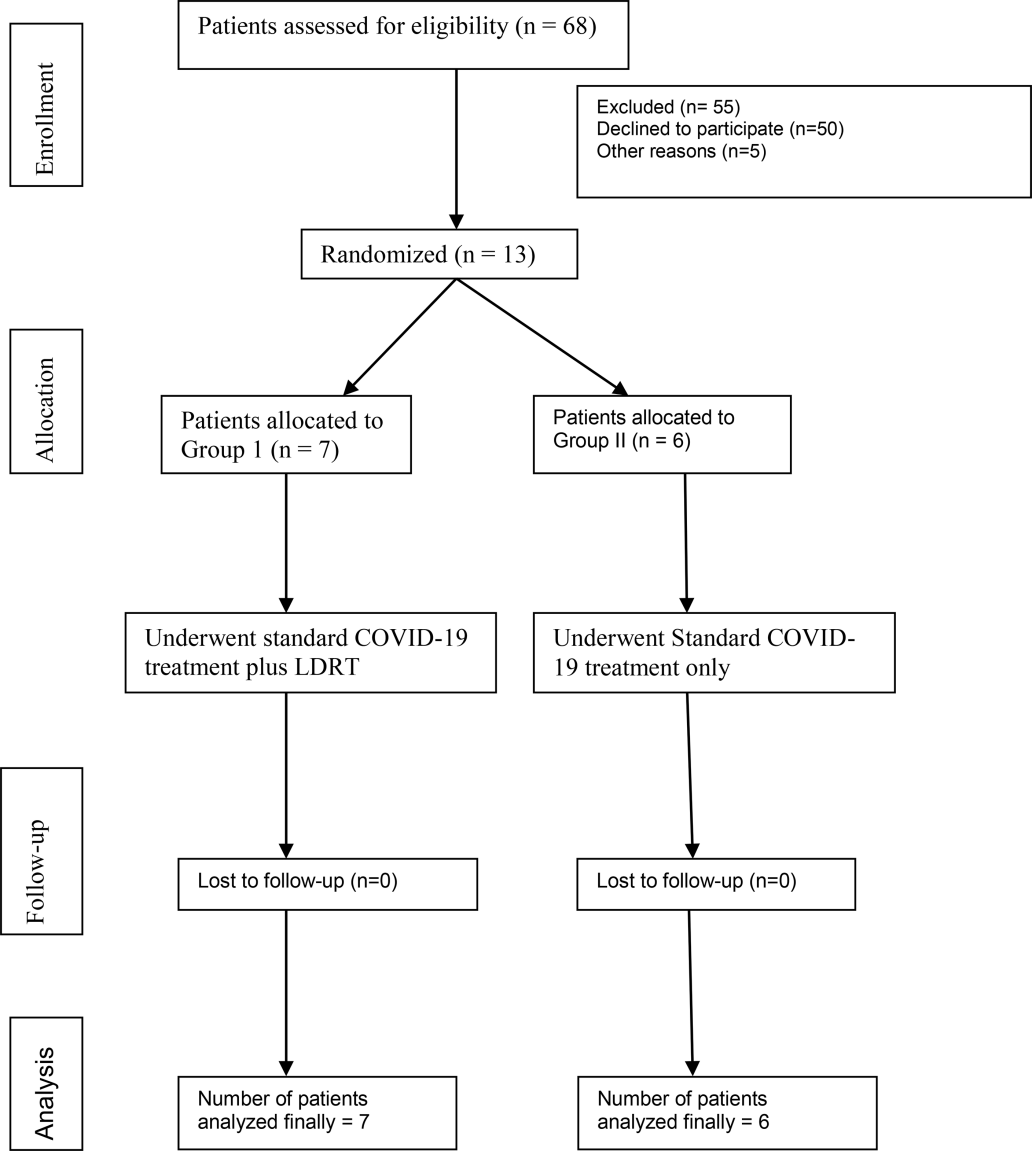
Consort flow chart.

## Introduction

Severe acute respiratory syndrome coronavirus 2 (SARS-CoV-2) which primarily affects the respiratory system results in a wide spectrum of symptoms, starting from mild cough to acute respiratory distress syndrome (ARDS) and multi organ failure. Epidemiological studies have observed that 6 to 10% of patients may develop a severe form of COVID-19 infection and require high quality intensive care unit (ICU) support including Mechanical ventilation (MV) (1). Mortality rates reported in patients with severe symptoms in the ICU may range from 25 to 65% with regional, institutional, and ethnic variations (2–4). However, treatment remains largely supportive till date and multiple clinical trials took place within the short span of time to evaluate the role of investigational therapies or ‘off-label’ medications such as remdesivir, therapeutic plasma exchange, convalescent plasma, and mesenchymal stem cell therapies to reduce the disease severity in COVID-19. These therapies are extensively administered worldwide after getting the emergency use authorization (EUA) but unfortunately failed to produce significant clinical recovery and reduction of mortality rate (5, 6). In this context, the role of low dose radiotherapy (LDRT) has re-emerged as a potent anti-inflammatory and immune modulator which can effectively reduce the severity of lung damage caused by the SARS-CoV-2. In this prospective, randomized clinical trial, we have studied the role of LDRT on the moderately severe COVID-19 pneumonia patients with the evaluation of clinical, biochemical, and radiological parameters.

The primary objective was to evaluate changes from moderate to severe grade of the disease in radiation and observation group with the National Early Warning Score (NEWS-2) at days 3, 7, and 14 (7, 8).

Secondary objectives of this trial include evaluation of changes in physiological parameters, namely, ratio between peripheral capillary oxygen saturation and fraction of inspired oxygen (SpO2/FiO2), changes in hematological and biochemical parameters such as Complete blood count (CBC), neutrophil to lymphocyte ratio (NLR), erythrocyte sedimentation rate (ESR), D-dimer, Lactate dehydrogenase (LDH), Ferritin, Interleukin-6 (IL-6), C-Reactive protein (CRP), arterial blood gases analysis (ABG), prothrombin time (PT), international normalized ratio (INR), D-dimer, IL-6 (interleukin-6) and changes in 25 point CT severity scoring on days 7 and 28. Radiation induced acute pulmonary toxicity is monitored on basis of the Common Terminology Criteria for Adverse Events (CTCAE v 5.0) scale (9).

## Methods

This prospective, double arm, interventional, randomized controlled trial was conducted from May 2021 to August 2021 at the All India Institute of Medical Sciences (AIIMS), Patna, India after obtaining clearance from the institutional ethical committee and registration with the Central Drugs standard control Organization India (ECR/1387/Ins/BR/2020) and the Clinical Trials Registry-India (CTRI/2021/06/033912, May 25, 2021). Thirteen patients aged between 18 and 70 years, with confirmed SARS-CoV2 infection by RT-PCR from nasopharyngeal swab and or broncho-alveolar lavage (BAL) were recruited. Patients had moderate disease as per the criteria laid by the Ministry of Health and Family Welfare (MOHFW), Government of India which included SpO2 <94% on Room Air and Respiratory rate >20 breaths per minute at room air with fewer than 10 days of symptom onset (10). Patients were randomly assigned to a radiotherapy (RT) arm and a non-RT arm with 1:1 ratio by computer-generated random numbers. All patients were receiving standard medication as per institute protocol for COVID-19 pneumonia. Patients with severe COVID-19 disease, hemodynamically unstable, autoimmune disease, under mechanical ventilation, and pregnancy were excluded from the study.

**Figure 1:**
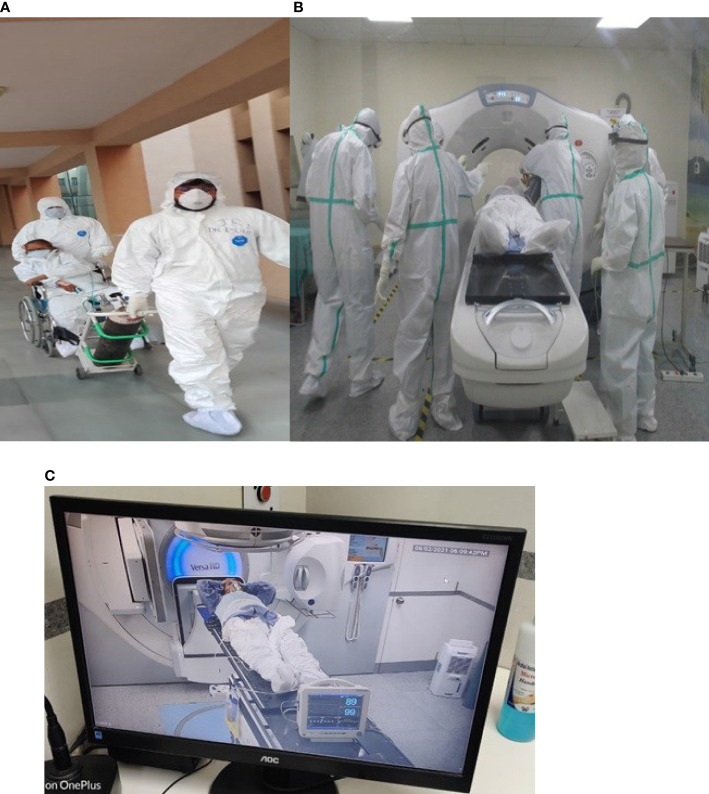
**(A)** (left) and **(B)** (right): Show the transfer and CT Simulation of the COVID 19 patients enrolled in our trial, maintaining proper personal protection. **(C)** Shows the continuous Surveillance and Monitoring of the patient during the delivery of LDRT.

The study was performed with strict compliance with institutional guidelines for infection prevention, namely, the use of personal protective equipment (PPE) by the entire team and the patients, dedicated patient transport corridor, and decontamination measures. Patients were transported from their dedicated COVID-19 wards to the department of radiotherapy under continuous monitoring by one physician and two paramedical personnel (**Figure 1A**) with supplementary oxygen support. After being shifted to the CT simulation room, a non-contrast CT scan of thorax with 5 mm slice thickness was done in supine position along with a 4-clamp thermoplastic immobilization cast and head rest (**Figure 1B**). Radiopaque markers were used to verify the coincidence of the isocentre marking on the cast with the isocentre defined in the planning CT scan. Average imaging parameters were calculated as 110 kVp and 130 mAs for most of the patients. The CT images were imported into the Monaco 5.11 treatment planning system. Window width and window level settings were adjusted to better visualize pulmonary tissue and window level/width equal to −400–600 HU/400–1, 600 HU was mostly applied before contouring. Clinical target volume (CTV) which included bilateral lung tissue was contoured promptly by the radiation oncologist with interpolation and manual edit. No organs at risk (OARs) were delineated during the same settings to reduce the overall treatment time. Heart, spinal cord, thyroid gland, head of humerus, liver, esophagus and breast tissue for female subjects were contoured later on for dosimetric purpose. No dose constraint was given to any other structure. The planning target volume (PTV) was generated by adding 0.7 mm margin around the CTV. The medical physics team planned a 3D conformal radiotherapy with anterior and posterior portals (AP/PA) with 6 MV and or 10 MV photon beams and equal weightage. The prescribed dose was 0.7 Gray (Gy) to bilateral lung fields for single fraction, with at least 95% of PTV receiving 95% of prescription dose. The overall required time since the entry of the patient and plan approval by the principal investigator was 15 min. The delivery of LDRT was executed with patient setup on Elekta Versa HD (Elekta, Stockholm, Sweden) under continuous monitoring with one of the in-room cameras directed to the monitor of the patient for remote surveillance (**Figure 1C**).

Patients in the non-RT arm were shifted to their respective COVID-19 ward just after CT simulation. The average time from entering to exit the radiation treatment area in the RT arm was 25 min whereas it was 10 min in the non-RT arm. The day of LDRT delivery or day of CT simulation was considered as day 1 and CT scan thorax was again done on days 7 and 28 for comparison of CT severity score of both groups. All the clinical records (general consciousness, temperature, pulse, blood pressure (BP), SpO2 at room air, SpO2 with oxygen, device used to maintain SpO2, FiO2, hematological and biochemical parameters were recorded on days 1, 3, 7, and 14 for both arms. These clinical and biochemical parameters were used to calculate the NEWS-2 score on respective days at a fixed 9:00 h.

The Statistical Package for Social Sciences version 25 (SPSS version 25.0) was used for the data analysis. Descriptive statistics was performed for clinical characteristics and demographic data. The primary endpoint was to compare the NEWS-2 score in both of the groups. Mean [ ± standard deviation (SD)] or median for continuous variables and frequency for categorical variables were reported. Continuous and the categorical data were compared using a 2-tailed Fisher’s exact test. A p-value <0.05 was considered statistically significant.

## Results

Investigators, residents of the department of radiotherapy, and primary physicians of the COVID-19 ward screened the patients who were admitted for management of COVID-19 at our institute and a total 68 patients with moderate COVID-19 pneumonia were screened for the trial. Patients and their family members were thoroughly explained about the possible side effects including the calculated attributable risk of second malignancy and the ‘off-label’ characteristic of LDRT in the treatment of COVID-19. Difficulty in obtaining consent was the major reason for poor accrual; however at the time of interim analysis we included seven patients in the RT arm and six patients in the non-RT arm (**Figure 1**). All enrolled patients had the history of less than 10 days since the onset of flu symptoms.

There were two female patients in the RT arm, whereas four female patients were in the non-RT arm. Approximately one-fourth of the patients had given history of smoking in both the arms. Two patients were diagnosed with diabetes mellitus (DM) during the hospital stay for management of COVID-19 while nine (69.2%) patients had known comorbidities (hypertension (HTN), DM, Asthma). Blood group A and Rh positivity were most commonly found in both arms (**Table 1**).

**Table 1:** Clinical characteristics of patients during admission and hospital stay.

		RT arm		Non-RT arm		p-value
		Count	N%	Count	N %	
Gender	Male	5	38.5%	2	15.4%	0.170
	Female	2	15.4%	4	30.8%	
Comorbidity	None	3	23.1%	1	7.7%	0.253
	DM	2	15.4%	1	7.7%	
	HTN + DM	1	7.7%	4	30.8%	
	HTN + Asthma	1	7.7%	0	0.0%	
Incidental diagnosed comorbidity	Yes	2	15.4%	0	0.0%	0.155
	No	5	38.5%	6	46.2%	
Blood group	A	4	30.8%	3	23.1%	0.107
	B	3	23.1%	0	0.0%	
	AB	0	0.0%	1	7.7%	
	O	0	0.0%	2	15.4%	
Rh	Positive	6	46.2%	4	30.8%	0.416
	Negative	1	7.7%	2	15.4%	
O2 Requirement day 1	Yes	7	53.8%	6	46.2%	–
	No	0	0.0%	0	0.0%	
O2 Requirement day 3	Yes	7	58.3%	5	41.7%	–
	No	0	0.0%	0	0.0%	
O2 Requirement day 7	Yes	3	25.0%	2	16.7%	0.921
	No	4	33.3%	3	25.0%	
O2 Requirement day 14	Yes	2	16.7%	0	0.0%	0.190
	No	5	41.7%	5	41.7%	

DM, diabetes mellitus; HTN, hypertension; A, blood group “A”; B, blood group “B”; AB, blood group “AB”; O, blood group “O”; O2, oxygen.

The median age of patients was 56 (52–68) years in the RT arm and 59 (50–69) years in the non-RT arm. The median days to get admitted after onset of symptoms were 5 (3–9 days). The median duration of hospital stay (14 days for both arms) and median duration on oxygen support (11 and 10 days in RT and Non RT arms respectively) were almost equal. The median SpO2 of patients at admission was 90% at room air. Other clinical parameters on days 1, 3, 7, and 14 are depicted in **Table 2**.

**Table 2:** Clinical parameters of patients on the day of LDRT and serial follow up.

	Radiotherapy arm (RT arm)					Non Radiotherapy arm (non-RT arm)				
	Mean	Median	Minimum	Maximum	SD	Mean	Median	Minimum	Maximum	SD
Age	58	56	52	68	6	60	59	50	69	7
Number of days on O2	13	11	4	23	7	11	10	6	24	7
Days of Hospital stay	18	14	4	51	15	15	14	6	30	8
SpO2 day 1	89	90	86	91	2	91	90	90	92	1
SBP day 1	130	131	108	154	17	121	116	110	142	13
DBP day 1	76	72	66	100	12	73	70	64	90	9
SpO2 day 3	92	92	90	94	1	92	92	91	92	0
SBP day 3	126	124	110	138	10	128	130	118	138	7
DBP day 3	65	72	24	82	19	75	76	64	82	8
SpO2 day 7	95	94	92	98	3	95	94	93	96	1
SBP day 7	127	128	118	136	7	125	122	120	132	5
DBP day 7	75	74	72	78	3	79	78	72	86	5
SpO2 day 14	94	96	80	98	6	97	97	96	98	1
SBP day 14	129	130	122	132	4	136	132	122	164	17
DBP day 14	77	78	70	84	5	81	80	76	88	5

SpO2, oxygen saturation at room air; SBP, systolic blood pressure; DBP, diastolic blood pressure; SD, standard deviation.

The dose prescribed and delivered to bilateral lungs was 0.7 Gy with 3D-CRT technique. The mean dose received by the OAR, namely, heart, esophagus, thyroid, liver, and spine were 67 cGy,64 cGy, 44 cGy, and 45 cGy, respectively. For female patients mean dose to right and left breast are 46 cGy and 51 cGy respectively.

The institutional management protocol for COVID-19 pneumonia was similar for both groups. All patients in our study received intravenous dexamethasone, enoxaparin, and azithromycin. Dose and duration of the medications were decided by the primary physician at the COVID ward. Remdesivir was given to two patients in the RT arm and four patients in the non-RT arm.

On day 1, the mean value of total leucocyte count (TLC) and neutrophil to lymphocyte ratio (NLR) were 9,160/ml and 16.895 in the RT arm and 12,630/ml and 8.722 in the non-RT arm respectively which showed no statistically significant difference at day 1. Other biochemical parameters, namely, d-dimer, PT, INR, CRP, LDH, IL-6, and ferritin similarly showed no statistically significant difference at day 1 (p >0.05) (**Table 3**).

**Table 3:** Comparison of different biochemical parameters on the day of LDRT and serial follow up.

		N	Mean	SD	95% Confidence Interval for Mean		Minimum	Maximum	P-value
					Lower Bound	Upper Bound			
NLR day 1	RT arm	7	16.895	13.600	−5.909	22.255	2.36	43.64	0.228
	Non-RT arm	6	8.722	8.307	−5.535	21.881	1.85	22.43	
D-dimer day 1	RT arm	7	1.415	0.977	0.512	2.319	0.45	3.43	0.377
	Non-RT arm	6	4.130	7.792	−4.047	12.307	0.49	20.00	
PT day 1	RT arm	7	13.014	1.820	11.330	14.698	11.02	16.50	0.332
	Non-RT arm	6	12.200	0.776	11.385	13.014	11.00	13.11	
INR day 1	RT arm	7	1.007	0.138	0.879	1.135	0.82	1.24	0.122
	Non-RT arm	6	0.898	0.084	0.810	0.986	0.75	0.97	
LDH day 1	RT arm	7	975.775	327.650	672.749	1,278.802	531.12	1411.00	0.249
	Non-RT arm	6	777.158	245.906	519.094	1,035.221	591.50	1202.50	
CRP day 1	RT arm	7	34.125	26.290	9.811	58.440	10.05	70.22	0.859
	Non-RT arm	6	37.588	41.940	−6.425	81.602	2.00	111.16	
Ferritin day 1	RT arm	7	764.864	343.729	446.967	1,082.761	365.10	1462.00	0.844
	Non-RT arm	6	718.035	492.046	201.664	1,234.405	307.50	1650.00	
IL-6 day 1	RT arm	7	16.942	20.238	−1.774	35.660	3.20	61.60	0.647
	Non-RT arm	6	24.316	35.412	−12.847	61.480	3.00	92.90	
NLR day 3	RT arm	7	11.268	5.661	−6.775	11.745	4.65	19.79	0.563
	Non-RT arm	5	8.783	8.824	−8.361	13.331	1.16	18.43	
D-dimer day 3	RT arm	7	2.805	4.405	−1.269	6.880	0.35	12.69	0.478
	Non-RT arm	5	1.300	1.140	−0.116	2.716	0.45	3.26	
PT day 3	RT arm	7	12.751	1.467	11.393	14.109	11.00	14.70	0.531
	Non-RT arm	5	12.280	0.783	11.307	13.252	11.31	13.48	
INR day 3	RT arm	7	0.958	0.092	0.872	1.044	0.85	1.10	0.294
	Non-RT arm	5	0.906	0.059	0.831	0.980	0.86	1.01	
LDH day 3	RT arm	7	752.205	187.854	578.469	925.941	435.92	1023.36	0.015
	Non-RT arm	5	496.792	45.994	439.682	553.901	427.31	552.70	
CRP day 3	RT arm	7	23.254	24.207	0.866	45.642	1.18	58.51	0.801
	Non-RT arm	5	26.764	21.323	0.287	53.241	5.52	62.49	
Ferritin day 3	RT arm	7	734.294	436.553	330.549	1,138.038	273.90	1650.00	0.592
	Non-RT arm	5	594.286	423.892	67.954	1,120.617	298.52	1331.00	
IL-6 day 3	RT arm	7	11.414	10.395	1.800	21.028	2.70	33.40	0.359
	Non-RT arm	5	25.860	38.515	−21.963	73.683	2.70	92.90	
NLR day 7	RT arm	7	20.153	13.788	−0.378	28.517	4.88	47.50	0.055
	Non-RT arm	5	6.084	4.626	1.066	27.072	2.62	14.00	
D-dimer day 7	RT arm	7	1.037	0.615	0.468	1.606	0.32	3.10	0.862
	Non-RT arm	5	1.074	1.117	−0.261	2.513	0.48	3.10	
PT day 7	RT arm	7	12.230	1.230	11.092	13.367	10.10	14.00	0.764
	Non-RT arm	5	12.026	0.961	10.832	13.219	11.10	13.48	
INR day 7	RT arm	7	0.864	0.102	0.769	0.958	0.72	.98	0.350
	Non-RT arm	5	0.914	0.055	0.845	0.982	0.87	1.01	
LDH day 7	RT arm	7	627.501	217.753	426.113	828.889	315.50	978.20	0.249
	Non-RT arm	5	485.800	163.264	283.079	688.520	321.80	716.16	
CRP day 7	RT arm	7	6.657	10.892	−3.417	16.731	1.02	31.22	0.149
	Non-RT arm	5	19.720	18.191	−2.867	42.307	2.21	50.18	
Ferritin day 7	RT arm	7	512.348	209.746	318.365	706.332	162.00	832.00	0.464
	Non-RT arm	5	661.470	462.616	87.055	1235.884	274.50	1225.00	
IL-6 day 7	RT arm	7	6.985	8.704	−1.064	15.036	2.70	26.30	0.789
	Non-RT arm	5	5.740	5.994	−1.703	13.183	2.70	16.40	
NLR day 14	RT arm	7	11.436	9.750	−3.288	16.960	4.50	32.41	0.163
	Non-RT arm	5	4.600	2.818	−2.289	15.961	1.77	9.11	
D-dimer day 14	RT arm	7	0.948	0.700	0.301	1.596	0.32	3.10	0.828
	Non-RT arm	5	1.058	1.009	−0.195	2.311	0.39	3.10	
PT day 14	RT arm	7	12.302	1.147	11.241	13.364	10.40	13.97	0.205
	Non-RT arm	5	11.446	0.966	10.246	12.645	10.20	12.83	
INR day 14	RT arm	7	0.917	0.068	0.853	0.980	0.84	1.04	0.105
	Non-RT arm	5	0.818	0.124	0.663	0.972	0.62	0.95	
LDH day 14	RT arm	7	584.004	199.388	399.600	768.407	300.20	788.15	0.500
	Non-RT arm	5	505.744	177.071	285.881	725.607	330.76	738.31	
CRP day 14	RT arm	7	14.432	19.035	−3.172	32.037	2.00	50.59	0.180
	Non-RT arm	5	37.957	37.435	−8.524	84.439	2.20	97.16	
Ferritin day 14	RT arm	7	407.805	175.436	245.553	570.057	175.70	755.80	0.995
	Non-RT arm	5	408.460	207.394	150.945	665.974	218.40	736.30	
IL-6 day 14	RT arm	7	4.300	4.252	0.367	8.232	2.00	13.90	0.974
	Non-RT arm	5	4.380	3.756	−0.284	9.044	2.70	11.10	

**Figure 2:**
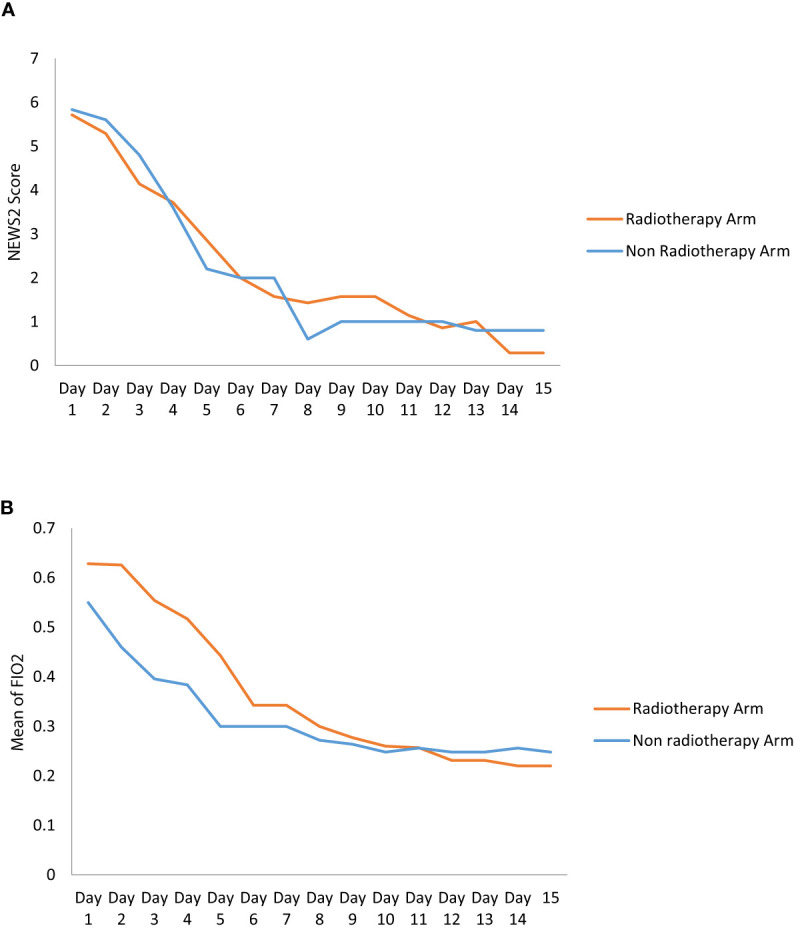
**(A)** The graph shows the change of NEWS2 Score in RT and Non RT arm on Day 1 and serial follow-up. **(B)** The Graph shows the change of mean of Fio2 in RT and Non RT arm on Day 1 and serial follow-up.

There was no statistically significant difference in NEWS-2 score in the RT and non-RT arms at day 3 (p = 0.360), day 7 (p = 0.617), and day 14 (p = 0.506). But there was a tendency of improvement of NEWS-2 score in the RT arm at day 3. There was a statistically significant difference in the improvement of FiO2 (p = 0.042) and SpO2/FiO2 (p = 0.028) at day 3 between the two arms, however this significant difference was gradually lost after the third day (**Figure 2** and **Table 4**). The 25 point CT severity score at enrollment had a median value of 16 and 18 in the RT and non-RT arm respectively. There were no statistically significant differences in the CT severity score in either of the two arms at day 1 (p = 0.325) and day 7 (p = 0.354).

**Table 4:** Oxygen parameters and NEWS-2 score on the day of LDRT and serial follow up.

		N	Mean	SD	95% Confidence Interval for Mean		Minimum	Maximum	P-value
					Lower Bound	Upper Bound			
O2 Flow day 1	RT arm	7	8.14	3.288	5.10	11.18	4	12	0.087
	Non-RT arm	6	5.50	1.049	4.40	6.60	4	7	
FiO2 day 1	RT arm	7	0.629	0.213	0.431	0.826	0.4	0.8	0.279
	Non-RT arm	6	0.517	0.116	0.394	0.639	0.4	0.7	
SpO2/FiO2 day 1	RT arm	7	158.928	58.791	−84.947	38.440	110.00	225.00	0.424
	Non-RT arm	6	182.182	37.903	−83.336	36.828	131.43	225.00	
NEWS-2 score day 1	RT arm	7	5.71	1.254	4.55	6.87	4	8	0.871
	Non-RT arm	6	5.83	1.329	4.44	7.23	4	7	
O2 Flow day 3	RT arm	7	6.00	2.828	3.38	8.62	2	10	0.088
	Non-RT arm	5	3.40	1.342	1.73	5.07	2	5	
FiO2 day 3	RT arm	7	0.540	0.202	0.352	0.727	0.28	0.80	0.042
	Non-RT arm	5	0.320	0.056	0.249	0.390	0.28	0.40	
SpO2/FiO2 day 3	RT arm	7	193.522	76.584	−186.672	−13.362	112.50	328.57	0.028
	Non-RT arm	5	293.539	47.234	−179.985	−20.049	230.00	328.57	
NEWS-2 score day 3	RT arm	7	4.14	1.345	2.90	5.39	2	6	0.360
	Non-RT arm	5	4.80	0.837	3.76	5.84	4	6	
O2 Flow day 7	RT arm	7	2.00	2.517	−0.33	4.33	0	5	0.266
	Non-RT arm	5	0.60	0.894	−0.51	1.71	0	2	
FiO2 day 7	RT arm	7	0.320	0.152	0.178	0.461	0.21	0.60	0.228
	Non-RT arm	5	0.230	0.030	0.191	0.268	0.21	0.28	
SpO2/FiO2 day 7	RT arm	7	351.598	138.779	−212.897	81.809	153.33	466.67	0.345
	Non-RT arm	5	417.142	54.778	−198.183	67.094	332.14	457.14	
NEWS-2 score day 7	RT arm	7	1.57	2.149	−0.42	3.56	0	5	0.617
	Non-RT arm	5	1.00	1.414	−0.76	2.76	0	3	
O2 Flow day 14	RT arm	7	2.57	5.593	−2.60	7.74	0	15	0.335
	Non-RT arm	5	0.00	0.000	0.00	0.00	0	0	
FiO2 day 14	RT arm	7	0.310	0.219	0.106	.5134	0.21	0.80	0.340
	Non-RT arm	5	0.210	0.000	0.210	.2100	0.21	0.21	
SpO2/FiO2 day 14	RT arm	7	385.948	140.308	−218.741	64.923	100.00	466.67	0.255
	Non-RT arm	5	462.857	3.984	−206.675	52.857	457.14	466.67	
NEWS-2 score day 14	RT arm	7	0.29	0.756	−0.41	0.98	0	2	0.506
	Non-RT arm	5	0.80	1.789	−1.42	3.02	0	4	

Radiation induced acute pulmonary toxicity was not observed in any of the patients in the radiation arm. One patient of the RT arm required MV after initial clinical recovery because of the development of sinonasal mucormycosis infection and the patient died in the ICU. A sudden cardiac death was reported in the non-RT arm.

## Discussion

Historically, X-ray therapy has been used for non-responsive pneumonia with promising results but had been abandoned with the advent of the newer antibiotic era (11–13). In the present scenario of COVID-19 pandemic, many studies have been re-initiated to evaluate the response of LDRT (0.5 to 1 Gray) to bilateral lungs and have reported rapid relief of respiratory symptoms in terms of decreased requirement of supplemental oxygenation, radiological, and biochemical responses (14–16). LDRT to the lung leads to the polarization of macrophages towards the anti-inflammatory M2 phenotype which helps to decrease the inflammatory process and possible evolvement into ARDS (17). It is therefore postulated that LDRT can also prevent cytokine storm by lymphocyte exhaustion and immune consumption. Reduction in adhesion of mononuclear cells to the endothelial cells and induction of apoptosis by LDRT may also lead to the modulation of inflammatory cell activity in COVID-19 (18, 19),. Hence LDRT delivered to both lungs in such patients is backed up by biological and clinical basis that justify its use as a possible therapeutic option (20). Many clinical studies have since evaluated the role of LDRT to lungs in COVID-19 pneumonia. Most of them are single arm studies and have documented feasibility, tolerability, and clinical benefit (16, 21, 22). However few studies such as that of Papachristofilou et al. demonstrated no benefit of LDRT to improve recovery in critically ill patients requiring MV for COVID-19 pneumonia (23). Available studies have also indicated the importance of the timing of LDRT to bilateral lung fields stating that there is a small window of opportunity after which radiation may not be adequately effective.

To the best of our knowledge this is the only randomized trial with moderate COVID-19 pneumonia patients and analyzed its role to halt the progression of a moderate disease to a severe one with NEWS-2 score. Although the primary physician was not blinded, we feel that the probable impact would have been minimal since all patients received the treatment according to the institutional protocol in the inpatient department as per their clinical severity. Moreover, the CT severity of both arms was scored by a single radiologist who was blinded to reduce bias. The other variables that were evaluated were laboratory tests which would not be impacted by the blinding process.

**Figure 3:**
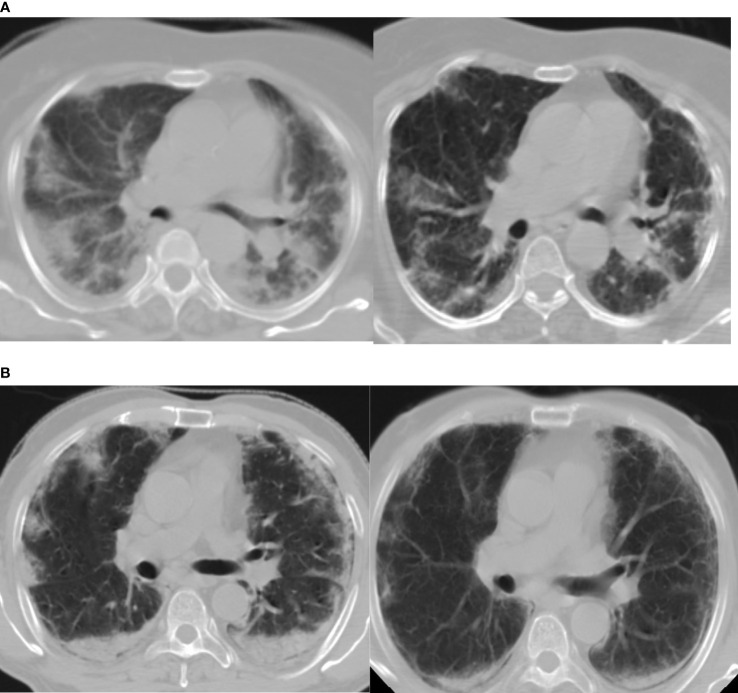
**(A, B)** Show marked radiological improvement on Day 7, compared to Day 1, of two different patients in the RT arm. Day 1(Left) D7(Right).

The dose for whole-lung LDRT was 0.7 Gy. According to a recent evaluation the lifetime attributable risk of cancer for a dose of 0.5 Gy for ages 20–80 can range from 0.29 to 1.7% for men and 0.5–4.9% for women (24). Cardiac events after 5 years of radiotherapy in the study by Darby et al. have shown a linear relationship with increasing dose by 7.4% per Gy (25). Taylor et al. have also shown a significant increase in cardiac mortality after 10 years of radiation to be 0.04% per Gy (26). The mean dose received by the heart in our study is approximately 0.67 Gy hence the probable effect of LDRT on the cardiovascular system needs to be monitored. We are following up the patients at 6 monthly intervals. The history of addictions like smoking in the background of co-morbidities increases the chance of attributable cancer and coronary heart disease. CT based planning with AP/PA portals helped in documenting the serial radiological response (**Figure 3**) with 25 points CT severity score and also to document the dosimetric profile of LDRT particularly to the adjacent organs such as the liver, heart, thyroid gland, spinal cord, and breasts for female patients. Therefore, we consider this trial as a unique opportunity to measure such dosimetric data which will serve as an important archive on longer follow up, if any recipient of LDRT develops any possible, attributable side effect in the lifetime.

The ratio of SpO2/FiO2 significantly decreased on the 3rd day in the LDRT arm which is similar to the other prospective studies but on days 7 and 14 of hospital stay, this significance was lost. A decrease in the level of biochemical markers of acute phase reactants (LDH, CRP, ferritin, and D-dimer) was seen in the RT arm and the non-RT arm. However, it failed to achieve clinical significance on comparison between the two arms.

The median duration of hospital stay is 16.8 days for the RT arm and 28 days in the non-RT arm. Although there was a trend towards early improvement, the difference did not show any statistical difference between the two arms after the third day. Lymphopenia was not seen in both arms suggesting no added deterioration with LDRT (27).

An interim analysis of our study failed to have any statistical improvement in either primary or secondary endpoints although the third day FiO2 was significantly better in the RT arm. Analyzing the two arms NEWS-2 score shows the RT arm continues to improve whereas the non-RT arm plateaus indicating that if more patients are added by virtue of completing the study might be able to reach statistical significance. Therefore, our results are not in complete agreement with the initial experiences reported from the non-randomized single-arm studies, which observed marked clinical, biochemical and radiographic improvement after LDRT for COVID-19 pneumonia.

### Limitations

The major limitations of this study include 1) Small sample size as it was extremely difficult to obtain proper consent of the patients and to judiciously select the moderately severe COVID-19 pneumonia patients. Differences were seen only for SpO2/FiO2 and LDH and only at day 3 while other clinical radiological and biochemical parameters failed to attain statistical significance due to very small size at the time of interim analysis. 2) Radiologist who prepared the 25 points CT severity score was blinded; however the treating physician and the team of radiation oncology were not blinded for this study. 3) The course of COVID-19 pneumonia and its clinical, biochemical, and radiological manifestations are itself heterogeneous and may vary between a wide range of spectrum. Similarly, patients with COVID-9 pneumonia respond differently to the standard protocol of the treatment. Hence it is expected that LDRT will not produce a similar response to all patients in the cohort, although we meticulously tried to include moderately affected patients.

### Conclusion

LDRT seems to have the potential to prevent moderate COVID-19 pneumonia from deteriorating to severe category. However, further randomized clinical trial with a greater number of such patients is warranted to establish the definitive role of LDRT in the management of COVID-19 pneumonia.

## Data Availability Statement

The raw data supporting the conclusions of this article will be made available by the authors, without undue reservation.

## Ethics Statement

The studies involving human participants were reviewed and approved by the All India Institute of Medical Sciences, Patna, India. The patients/participants provided their written informed consent to participate in this study.

## Author Contributions

PS: Conceptualization, data curation, funding acquisition, investigation, project administration, writing - review and editing. AM: Conceptualization, data curation, investigation, writing - review and editing. DS: Conceptualization, data curation, investigation, software, writing - review and editing. SK: Conceptualization, formal analysis, validation, writing - review and editing. AK: Conceptualization, formal analysis, validation, writing - review and editing. AR: Investion, resources. RR: Investigation, resources. MV: Investigation, resources. DKR: Conceptualization, investigation. DB: Conceptualization, investigation. AS: Software validation. ASi: Investigation, software. RS: Investigation, resources. ASa: Investigation, resources. AT: Investigation, resources. AKB: Investigation, supervision, SC: Investigation, supervision. RK: Investigation, resources, PK: Investigation, resources, BM: Investigation, resources. AR: Statistical analysis. All authors listed have made a substantial, direct, and intellectual contribution to the work and approved it for publication.

## Funding

Intramural funding with no I- 27/621 was provided from All India Institute of Medical Sciences Patna.

## Conflict of Interest

The authors declare that the research was conducted in the absence of any commercial or financial relationships that could be construed as a potential conflict of interest.

## Publisher’s Note

All claims expressed in this article are solely those of the authors and do not necessarily represent those of their affiliated organizations, or those of the publisher, the editors and the reviewers. Any product that may be evaluated in this article, or claim that may be made by its manufacturer, is not guaranteed or endorsed by the publisher.
